# Simultaneous enhancement of the beta–exo synergism and exo–exo synergism in *Trichoderma reesei* cellulase to increase the cellulose degrading capability

**DOI:** 10.1186/s12934-019-1060-x

**Published:** 2019-01-18

**Authors:** Hao Fang, Runze Zhao, Chaofeng Li, Chen Zhao

**Affiliations:** 1 College of Life Sciences, Northwest A&F University, 22 Xinong Road, Yangling, 712100 Shaanxi China; 2 Biomass Energy Center for Arid and Semi-arid Lands, Northwest A&F University, 22 Xinong Road, Yangling, 712100 Shaanxi China

**Keywords:** *Trichoderma reesei*, Cellulase, β-Glucosidase, Cellobiohydrolase, Synergism

## Abstract

**Background:**

Cellulase is the one of the largest contributors to the high production costs of the lignocellulose-based biorefineries. As the most widely used cellulase producer, *Trichoderma reesei* has two weaknesses, deficiencies in β-glucosidase and cellobiohydrolase II. This work aimed at solving this problem by simultaneous enhancement of the beta–exo synergism and exo–exo synergism in *T. reesei* cellulase to increase the cellulose degrading capability, i.e. enhanced co-expression of the β-glucosidase gene the cellobiohydrolase II gene of *T. reesei*.

**Results:**

Enhanced co-expression of the β-glucosidase gene and the cellobiohydrolase II gene in *T. reesei* using the strong promoter *Pcbh1* was found successful in overcoming the two weaknesses. Filter paper activities of *T. reesei* cellulase were greatly elevated, which were 7.21 ± 0.45 (E7, *Aabgl1* and *Trcbh2*) and 7.69 ± 0.42 (F6, *Anbgl1* and *Trcbh2*) FPIU/mL. They were much higher than that of the parental strain Rut-C30, 2.45 ± 0.36 FPIU/mL. Enzymatic hydrolysis yields were also improved, from 67.22 ± 1.61% by Rut-C30 cellulase to 87.98 ± 0.65% by E7 cellulase and 86.50 ± 1.01% by F6 cellulase. The substrate loading for 1 g glucose release from SECS were decreased, from 2.9637 g SECS using Rut-C30 cellulase to 2.0291 g SECS using E7 cellulase and 2.0573 g SECS using F6 cellulase. As a result, the efficiency of the process from SECS to glucose was substantially improved.

**Conclusions:**

Enhanced co-expression of the β-glucosidase gene and the cellobiohydrolase II gene in *T. reesei* using the strong promoter *Pcbh1* in *T. reesei* was proven triumphal in the simultaneous enhancement of the beta–exo synergism and exo–exo synergism in *T. reesei* cellulase. This strategy also improved the cellulase production, enzymatic hydrolysis yield and the efficiency of the process from SECS to glucose in the context of on-site cellulase production. This work is a commendable attempt in the cellulase composition optimization at the transcriptional level.

## Background

Cellulase is one of the hindrances to the commercialization of lignocellulose-based biorefineries [1–3], because of the fact that cellulase preparations in market are too expensive. Therefore, increasing the productivity and the specific activity of cellulase, improving the performance in the enzymatic hydrolysis of lignocelluloses, and reducing the production cost and the application cost of cellulase are key to making the biorefineries economically competitive.

As one of the most widely used cellulase producers in industry, *Trichoderma reesei* has the most robust cellulase and the production level was up to 100 g/L [3–5]. *T. reesei* cellulase has three categories of components: cellobiohydrolases (CBH: EC 3.2.1.91), which liberate cellobiose molecules from the reducing and nonreducing ends of a cellulose chain; endoglucanases (EG: EC 3.2.1.4), which attack internal bonds in the cellulose chain; and β-glucosidases (BG: EC 3.2.1.21), which hydrolyze cellobiose into glucose [1, 4, 6, 7]. Only the complete and balanced composition of the cellulase can accomplish the efficient enzymatic hydrolysis of cellulosic materials [7–10]. The components of the cellulase collaborate for the enzymatic hydrolysis of cellulose. A concept capable of describing this kind of co-work is synergism [4, 11, 12].

According to the optimal composition of the cellulase both in theory and in practice, *T. reesei* cellulase must have balanced composition to lead to utmost synergism and superb performance in enzymatic hydrolysis of lignocellulose [4, 8, 13]. Though *T. reesei* is one of the most robust cellulase-producers [14–16], the composition of its cellulase is not sufficiently excellent for various applications [4, 8, 17–19]. The two extensively reported weaknesses are the deficiencies of BG and CBH II [1, 4, 6, 7, 17, 20–22].

Many approaches had been proposed to overcome BG deficiency: direct addition of external BG into cellulase mixture [23–25]; co-cultivation with *Aspergillus* strains [1, 17, 26]; and genetic engineering of *T. reesei* for enhancing BG [20–22]. CBH II deficiency had been overcome by adding external CBH II into cellulase mixture or enhancing CBH II production by *T. reesei* [6, 7, 27]. However, these researches just tackled one of the weaknesses. The previous work succeeded to solve the two problems by combining genetic engineering and co-cultivation [4]. Thus, why not genetically engineer *T. reesei* for the simultaneous enhancement of beta–exo synergism and exo–exo synergism to overcome the two weaknesses once and for all?

In this work, three different BG genes from *T. reesei*, *Aspergillus aculeatus* and *Aspergillus niger* were placed under the control of *T. reesei*’s *Pcbh1* strong promoter respectively, and then introduced into *T. reesei*. The resulted *T. reesei* transformants were compared in the fermentation for cellulase production. Then the three strong expression cassettes were linked to the strong expression cassette of *T. reesei cbh2* ditto using *Pcbh1*. These strong expression cassettes of BG genes and CBH II were introduced into *T. reesei* respectively, and the resultant transformants were compared in the fermentation for cellulase production. The cellulases from all the *T. reesei* transformants were compared in the enzymatic hydrolysis to select the most potent cellulase and its producer, and more importantly to prove which combination of BG and CBH II expression cassettes is the best for the simultaneous enhancement of the beta–exo synergism and exo–exo synergism in *T. reesei* cellulase.

## Materials and methods

### Genes and vector constructions

The BG genes including *bgl1* (GenBank accession: D64088) from *A. aculeatus* (abbreviated as *Aabgl1*) and *bgl1* (GenBank accession: KJ739789) from *A. niger* (abbreviated as *Anbgl1*) were codon-optimized according to the *T. reesei*’s codon preference [28]. The genes, *bgl1* (GenBank accession: U09580) and *cbh2* (GenBank accession: M16190) from *T. reesei* which were abbreviated as *Trbgl1* and *Trcbh2*, do not need codon-optimizing because they were expressed homologously. All these gene sequences, whose signal peptide sequences and introns were deleted, were synthesized by Generay Biotech (Shanghai, China).

**Fig. 1:**
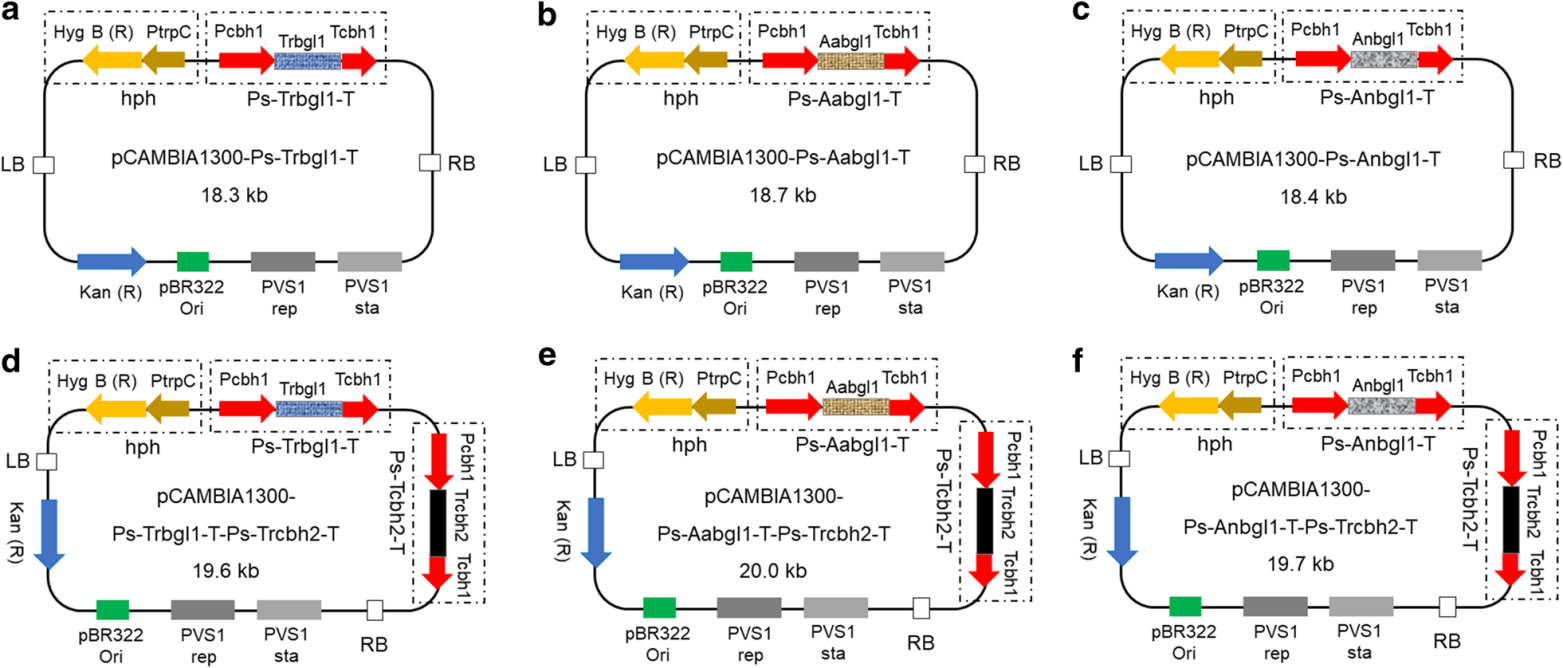
**a** The recombinant plasmid pCAMBIA1300-Ps-Trbgl1-T with the strong expression cassette of *T. reesei bgl1* gene; **b** the recombinant plasmid pCAMBIA1300-Ps-Aabgl1-T with the strong expression cassette of *A. aculeatus bgl1* gene; **c** the recombinant plasmid pCAMBIA1300-Ps-Anbgl1-T with the strong expression cassette of *A. niger bgl1* gene; **d** the recombinant plasmid pCAMBIA1300-Ps-Trbgl1-T-Ps-Trcbh2-T with the strong expression cassettes of *T. reesei bgl1* gene and *T. reesei cbh2* gene; **e** the recombinant plasmid pCAMBIA1300-Ps-Aabgl1-T-Ps-Trcbh2-T with the strong expression cassettes of *A. aculeatus bgl1* gene and *T. reesei cbh2* gene; **f** the recombinant plasmid pCAMBIA1300-Ps-Anbgl1-T-Ps-Trcbh2-T with the strong expression cassettes of *A. niger bgl1* gene and *T. reesei cbh2* gene. The hygromycin B resistance marker (PtrpC-hygB) and the kanamycin resistance marker (Kan) in the plasmid were used for selection of fungal and bacterial transformants, respectively

By subcloning, these sequences were linked to the promoter *Pcbh1* with the signal peptide sequence of *cbh1* and the terminator *Tcbh1* from *T. reesei* to form the strong expression cassettes, which were then linked to the plasmid pCAMBIA1300 to form the final vectors [4, 7], as shown in Fig. 1.

### Microorganisms

*Escherichia coli* DH5α was used for plasmid constructions and propagations. *Agrobacterium tumefaciens* AGL-1was used for transformation of *T. reesei*. *T. reesei* Rut-C30 was used as host for genetic engineering [4, 7]. All these microorganisms are preserved in Biomass Energy Center for Arid and Semi-arid Lands, Northwest A&F University.

### Lignocellulosic material and pretreatment

The lignocellulosic material used here was corn stover, which was collected from Kaifeng City, Henan Province in the year of 2015. It was sized down to a length of 5–10 cm, rinsed with tap water to remove sticky clay, and then air-dried for short term storage. Steam explosion was used as the pretreatment method [1, 2, 4, 29]. After the pretreatment, the steam exploded corn stover (SECS) was washed with distilled water until neutral pH to remove inhibitors.

The composition of the washed SECS was as follows (dry biomass): glucan 53.2%, xylan 6.9%, lignin 22.7%, ash 11.2%, and others 6.0%.

### Media

Luria–Bertani (LB) medium was used to cultivate *E. coli* and *A. tumefaciens*, which had the following composition (g/L): tryptone 10, yeast extract 5, NaCl 10. The LB medium was autoclaved at 121 °C for 20 min. For LB agar slants or plates, 1.5 g agar was added before autoclave. Rifamycin and/or Kanamycin were added when selective LB media were prepared.

Induction medium (IM) for *A. tumefaciens*-mediated transformation (AMT): add 0.8 mL of K-buffer, 20 mL of MN buffer, 1 mL of 1% (w/v) CaCl_2_∙2H_2_O, 10 mL of 0.01% (w/v) FeSO_4_, 5 mL of trace elements for IM medium, 2.5 mL of 20% (w/v) NH_4_NO_3_, 10 mL of 50% (vol/vol) glycerol, 40 mL of 1 M MES, pH 5.5, and 10 mL of 20% (w/v) glucose to 900.7 mL of sterilized water to make up 1 L of liquid IM [30].

The composition of the seed medium for pre-culturing *T. reesei* was as follows: 10 g/L glucose, 1 g/L peptone, 5 mL Mandels nutrient salts solution [31], 2.5 mL citrate buffer (1 mol/L), 0.05 mL Mandels trace elements solution [31], 0.1 g/L Tween 80. This seed medium was autoclaved at 121 °C for 20 min.

The fermentation medium for cellulase production by *T. reesei* was composed of 30 g/L SECS (dry biomass), 1 g/L glucose, 6 g/L (NH_4_)_2_SO_4_, 2.0 g/L KH_2_PO_4_, 0.3 g/L CaCl_2_, 0.3 g/L MgSO_4_, 0.005 g/L FeSO_4_, 0.0016 g/L MnSO_4_, 0.0014 g/L ZnSO_4_ and 0.0037 g/L CoCl_2_. The initial pH was adjusted to 4.8 with citrate buffer. This fermentation medium was autoclaved at 121 °C for 30 min.

The medium (MCC agar plates) for the second step of *T. reesei* transformant screening was composed of 20 g/L microcrystalline cellulose (MCC), 5 g/L (NH_4_)_2_SO_4_, 15 g/L KH_2_PO_4_, 0.8 g/L MgSO_4_, 0.6 g/L CaC1_2_, 0.005 g/L FeSO_4_·7H_2_O, 0.0016 g/L MnSO_4_·H_2_O, 0.0014 g/L ZnSO_4_·7H_2_O, 0.0037 g/L CoCl_2_·6H_2_O, 20 g/L agar. The initial pH was adjusted to 4.8 with citrate buffer. This medium was autoclaved at 121 °C for 20 min. Hygromycin B was added to a final concentration of 100 mg/L. Then it was poured into Petri dishes to form MCC agar plates [4, 7].

All the chemicals used in preparing theses media were purchased from Sinopharm Chemical Reagent Co. Ltd., Shanghai, China. All the antibiotics used here were bought from Sangon Biotech, Shanghai, China.

### *Agrobacterium tumefaciens*-mediated transformation of *T. reesei* and transformant screening

AMT of *T. reesei* and transformant screening was conducted according to the reported protocols with slight modifications [4, 7, 30]. The recombinant vector was transformed into *A. tumefaciens*. Then *A. tumefaciens* transformants harboring the vectors (Fig. 1) were co-cultured with *T. reesei* spore germlings in liquid IM medium and then the co-culture mixtures were spread on the nitrocellulose filters tiled on the IM agar plates. They were incubated at 24–25 °C for 2–3 days. Subsequently, the nitrocellulose filters carrying green spores with potential *T. reesei* transformants and remaining *A. tumefaciens* were reversely paved on the PDA agar plates containing 200 μM cefotaxime to kill *A. tumefaciens* cells and 100 μg/mL hygromycin B to select potential *T. reesei* transformants. This was the first step of transformant screening. *T. reesei* transformants appeared on the selective PDA agar plates were transferred to the MCC agar plates for the second step of transformant screening where transformant’s growth rate was used as the selection criterium [4, 7]. The fast-growing *T. reesei* transformants were chosen and compared in the following experiments.

### Fermentation for cellulase production

*Trichoderma reesei* must be pre-cultured for 36 h to reach a certain amount of biomass for the subsequent fermentation. The pre-culture was operated at 30 °C with a shaking of 170 rpm in 250 mL Erlenmeyer flasks with 50 mL of the seed medium. Then 5 mL (10% v/v) of the pre-cultures were inoculated into the fermentation medium for cellulase production. The fermentation was carried out in shaking incubator (170 rpm) at 30 °C on the first day and 28 °C since then. Sampling was conducted once a day during the fermentation process for analyses.

### Enzymatic hydrolysis of steam exploded corn stover

In order to compare the cellulases from different *T. reesei* strains, the enzymatic hydrolysis of SECS was implemented in 250 mL Erlenmeyer flasks with a working volume 50 mL containing 2.5 mL 1 M citrate buffer solution (for final pH 4.8), 100 g/L SECS (dry material), 25 FPIU/g glucan the cellulase harvested after 5 days fermentation, and a supplementary amount of water to make up 50 mL. After the cellulase being added, these flasks were incubated in an orbital shaker (140 rpm) at 50 °C for 48 h.

### Analytical methods

Filter paper activity (FPA) was assayed using the standard method recommended by the International Union of Pure and Applied Chemistry (IUPAC) [32], which measures the total amount of the reducing sugars released from 50 mg Whatman No.1 filter paper (1 × 6 cm strip) in 60 min reaction. One International Unit of FPA (FPIU) was defined as the amount of enzyme needed for releasing 1 μmol reducing sugars in 1 min.

β-Glucosidase activity (BGA) was determined using the standard method recommended by IUPAC [32] but the substrate was *ρ*NPG (*ρ*-nitrophenyl-β-d-1,4-glucopiranoside) (Sigma-Aldrich, St. Louis, MO, USA). The amount of *ρ*-nitrophenol formed in 10 min of the reaction was quantified spectrophotometrically at 400 nm. One International Unit of BGA (IU) was defined as the amount of enzyme required for releasing 1 μmol of *ρ*-nitrophenol in 1 min.

The methods for assaying cellobiohydrolase activity (CBA) and endoglucanase activity (EGA) were modified from the FPA measurement method [32]. Microcrystalline cellulose PH101 and carboxymethyl cellulose (Sigma-Aldrich, St. Louis, MO, USA) in terms of 1% (w/v) suspensions were used as the substrates for the reactions with the duration of 30 min. One Unit (1 U) of CBA or EGA was defined as the amount of enzyme needed for producing 1 mg reducing sugars in 1 h.

Glucose released from the enzymatic hydrolysis of SECS was quantified using a HPLC system (Agilent 1100) equipped with Bio-Rad Aminex HPX-87H (300 mm × 7.8 mm). Deionized and degassed water was used as the mobile phase at a low rate of 0.6 mL/min. The column temperature was maintained at 55 °C. The eluate was detected by a refractive index detector.

The enzymatic hydrolysis yield of SECS was calculated according to Eq. (1):

1 $$ {\text{Yield }}\left( \% \right) \, = {\text{ glucose }}\left( {\text{g}} \right) \, \times \, 0. 9 { } \times { 1}00/{\text{ glucan in SECS }}\left( {\text{g}} \right). $$

At least triplicates were used in all analyses and data are presented in the form of mean ± standard deviation.

## Results and discussion

### Enhanced expression of different *bgl1* genes

**Fig. 2:**
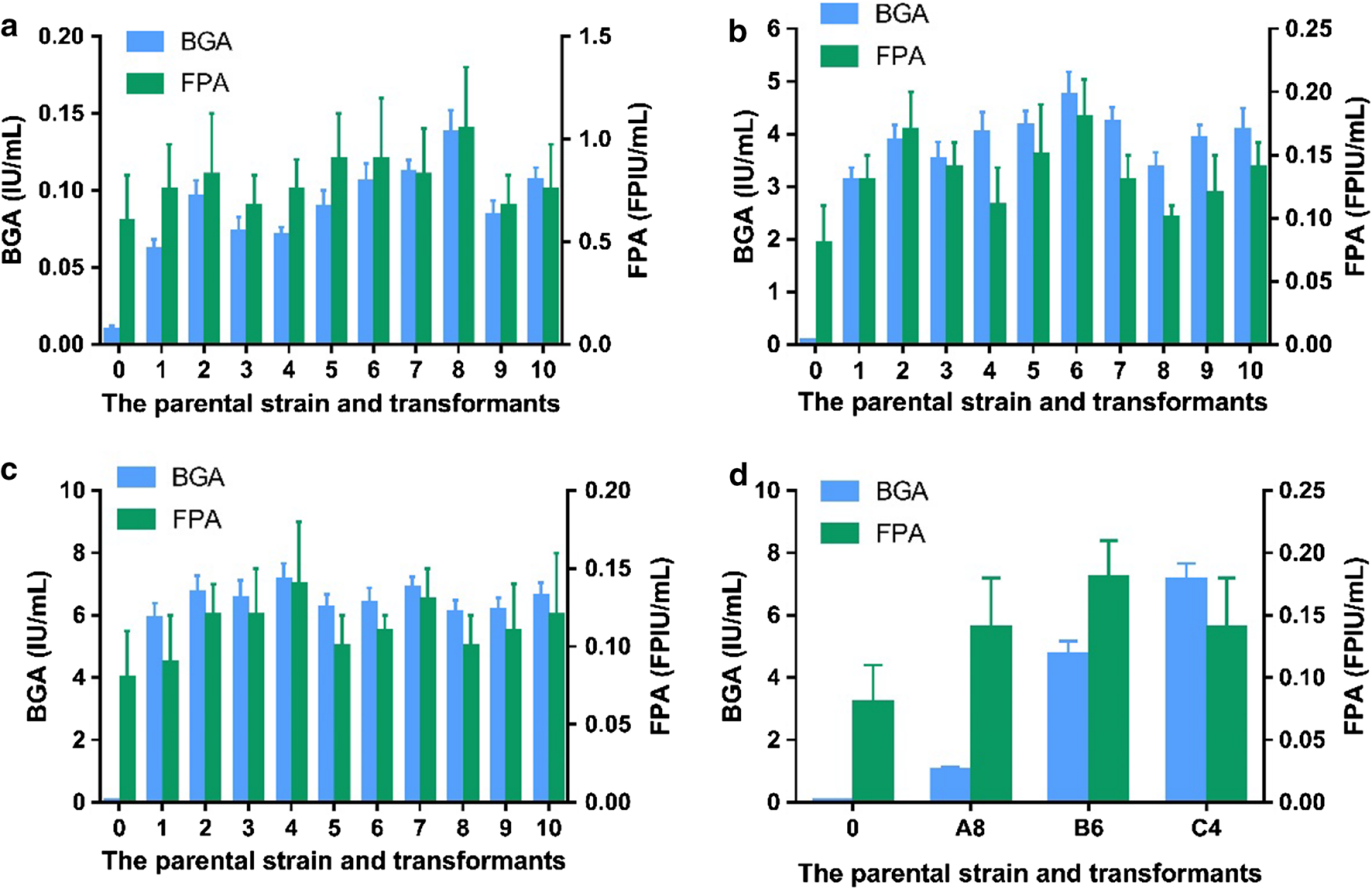
**a** BGAs and FPAs of the 10 fastest-growing *T. reesei* transformants (1 to 10) harboring the strong expression cassette of *T. reesei bgl1* gene and the parental strain Rut-C30 (0); **b** BGAs and FPAs of the 10 fastest-growing *T. reesei* transformants (1 to 10) harboring the strong expression cassette of *A. aculeatus bgl1* gene and the parental strain Rut-C30 (0); **c** BGAs and FPAs of the 10 fastest-growing *T. reesei* transformants (1 to 10) harboring the strong expression cassette of *A. niger bgl1* gene and the parental strain Rut-C30 (0); **d** Comparison of the top *T. reesei* transformants A8, B6 and C4 from the transformants screening presented in **a**–**c**, using the parental strain Rut-C30 as the control (0)

The recombinant vector pCAMBIA1300-Ps-Trbgl1-T (Fig. 1a) carrying the strong cassette of *T. reesei bgl1* gene was transformed to *T. reesei* by AMT, producing 203 transformants. pCAMBIA1300 is the backbone, Ps the strong promoter of the *T. reesei cbh1* gene and its signal peptide sequence, Trbgl1 the *T. reesei bgl1* gene, and T the terminator of the *T. reesei cbh1* gene. After the two steps of transformant screening, 10 fastest-growing transformants were selected to compare their BGAs and FPAs, as presented in Fig. 2a. The number 0 stands for the parental strain, i.e. *T. reesei* Rut-C30, and the numbers 1 to 10 are the *T. reesei* transformants. The ten *T. reesei* transformants A1–A10, cultured on the PDA agar plates without hygromycin B and were passed on from generation to generation for 10 times, were verified by genome PCR for the stable existence of the strong cassette of *T. reesei bgl1* gene in their chromosomal DNA (data not shown).

It was found that the BGAs of the 10 fastest-growing transformants were obviously improved while their FPAs were slightly higher than the parental strain, which in line with the previous report [21]. Though the increase of the BGAs here were larger than those in the previous report, the FPAs were nearly the same. The possible reasons included that different *T. reesei* transformation method and different BGA assay method were used here.

Among the 10 fastest-growing transformants, the 8th transformant (named as A8) had the highest BGA and FPA which were 1.03 ± 0.11 IU/mL and 0.14 ± 0.04 FPIU/mL after 48 h fermentation. They were 14.71 and 1.75 times higher than those of the parental strain, which were 0.07 ± 0.02 IU/mL and 0.08 ± 0.03 FPIU/mL after 48 h fermentation.

The recombinant vector pCAMBIA1300-Ps-Aabgl1-T (Fig. 1b) carrying the strong cassette of *A. aculeatus bgl1* gene was transformed to *T. reesei*, producing 236 transformants. pCAMBIA1300 is the backbone, Ps the strong promoter of the *T. reesei cbh1* gene and its signal peptide sequence, Aabgl1 the *A. aculeatus bgl1* gene, and T the terminator of the *T. reesei cbh1* gene. After the two steps of transformant screening, 10 fastest-growing transformants were picked off to compare their BGAs and FPAs which are shown in Fig. 2b. The number 0 is *T. reesei* Rut-C30, and the numbers 1 to 10 are the *T. reesei* transformants. The ten *T. reesei* transformants B1–B10, cultured on the PDA agar plates without hygromycin B and were passed on from generation to generation for 10 times, were verified by genome PCR for the stable existence of the strong cassette of *A. aculeatus bgl1* gene in their chromosomal DNA (data not shown).

As shown in Fig. 2b, the BGAs of the 10 fastest-growing transformants were greatly improved and their FPAs were higher than Rut-C30. The results resemble the previous work, especially for BGA increasement, but FPA increasement here was a little higher [20]. Among the 10 fastest-growing transformants, the 6th transformant (named as B6) had the highest BGA and FPA which were 4.74 ± 0.44 IU/mL and 0.18 ± 0.03 FPIU/mL after 48 h fermentation. They were 67.71 and 2.25 times higher than those of the parental strain whose BGA and FPA were 0.07 ± 0.02 IU/mL and 0.08 ± 0.03 FPIU/mL after 48 h fermentation.

The recombinant vector pCAMBIA1300-Ps-Anbgl1-T (Fig. 1c) carrying the strong cassette of *A. niger bgl1* gene was transformed to *T. reesei*, producing 217 transformants. pCAMBIA1300 is the backbone, Ps the strong promoter of the *T. reesei cbh1* gene and its signal peptide sequence, Anbgl1 the *A. niger bgl1* gene, and T the terminator of the *T. reesei cbh1* gene. After the two steps of transformant screening, 10 fastest-growing transformants were selected to compare their BGAs and FPAs which are presented in Fig. 2c. The number 0 is *T. reesei* Rut-C30, and the numbers 1 to 10 are the *T. reesei* transformants. The ten *T. reesei* transformants C1–C10, cultured on the PDA agar plates without hygromycin B and were passed on from generation to generation for 10 times, were verified by genome PCR for the stable existence of the strong cassette of *A. niger bgl1* gene in their chromosomal DNA (data not shown).

It was also found in Fig. 2c that the BGAs of the 10 fastest-growing transformants were prodigiously improved and their FPAs were higher than Rut-C30. The results are almost the same as the previous report [22]. Among the 10 fastest-growing transformants, the 4th transformant (named as C4) had the highest BGA and FPA which were 7.14 ± 0.52 IU/mL and 0.14 ± 0.04 FPIU/mL after 48 h fermentation. They were 102.00 and 1.75 times higher than those of the parental strain, 0.07 ± 0.02 IU/mL and 0.08 ± 0.03 FPIU/mL after 48 h fermentation.

Then the BGAs and FPAs of the outperformed *T. reesei* transformants (A8, B6, C4) were compared, as shown in Fig. 2d. It was found that the transformant C4 had the highest, much higher BGA than B6 and A8. Next in line is the transformant B6. The difference in specific enzymatic activity of these three β-glucosidases maybe lead to the different results. The previous cases [20–22] were repeated and compared here in the same research.

The possible reason for the large BGA increase but small FPA increase is that FPA determination method assayed the total amounts of reducing sugars but not merely glucose [32, 33]. In addition, though β-glucosidases play an important role in enzymatic hydrolysis of lignocelluloses [1, 17, 23], the contribution of β-glucosidases to overall yield is not so large [13, 34]. This means the increase of β-glucosidase content in cellulase mixture from 1 to 3% or 4% is sufficient for efficient enzymatic hydrolysis [13, 34] and the overproduction of β-glucosidase is a kind of waste which has been already beyond the demand of real-world applications. The following experiment of enzymatic hydrolysis would also prove this statement.

### Cellulase production with enhanced BGAs and the application in enzymatic hydrolysis

**Fig. 3:**
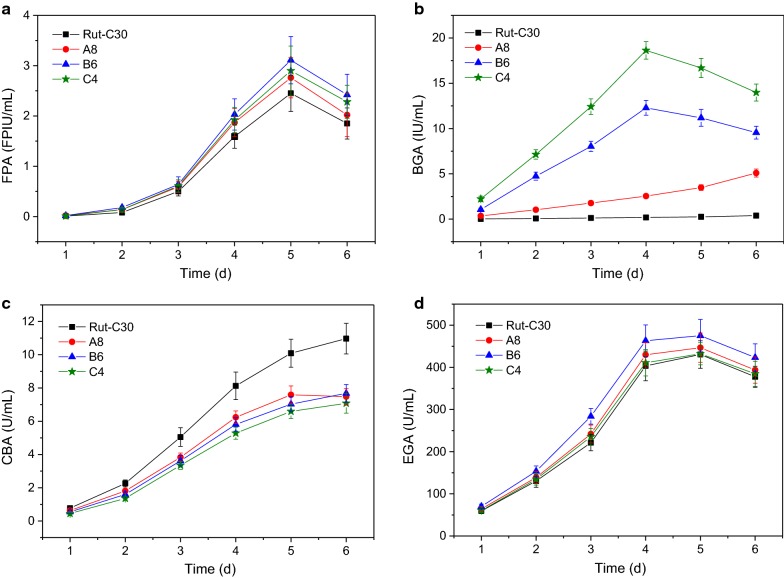
FPAs (**a**), BGAs (**b**), CBAs (**c**) and EGAs (**d**) in the fermentation broths obtained as a function of time from the *T. reesei* transformants A8, B6, C4, and parental strain Rut-C30 conducted in the 50 mL fermentation medium in 250 mL Erlenmeyer flasks incubated in incubatory shakers (170 rpm) at 30 °C on the first day and 28 °C since then

The time courses of the cellulase production by the *T. reesei* transformants A8, B6 and C4, as well as the control Rut-C30 are shown in Fig. 3. It was found in Fig. 3a that the FPAs of the transformants A8, B6 and C4 were not largely improved during the fermentation process as the BGAs were enhanced. Besides the explanations given above, the small decrease in CBAs (Fig. 3c) offset the increase caused by the elevated production of BGAs because CBA has very grand contribution [13, 34]. The decrease in CBAs should be attributable to that the energy of the strong promoter *Pcbh1* was partially diverted to the enhanced expression of the *bgl1* gene, i.e. transcription titration effect [35].

No significant difference was found in EGAs (Fig. 3d). This is because the genetic engineering strategy of this work did not touch endoglucanases. The minor difference in BGAs was probably caused by the interfering effect of the BGA and CBA changes and the errors from fermentation and enzymatic activity assay.

It is noteworthy that though the BGAs of the transformants B6 and C4 were much higher than those of A8 and Rut-C30, they declined faster than the latter ones. They peaked on day 4 but the BGAs of A8 and Rut-C30 continued to increase during the fermentation process. This may be because the β-glucosidase genes expressed in B6 and C4 were from *A. aculeatus* and *A. niger*, i.e. heterologous, making them unstable because heterologous proteins are much easier to be degraded by the proteases from the host [35, 36].

The cellulases produced by the strains A8, B6, C4 and Rut-C30 were harvested after 5 days fermentation because their FPAs, which reveal the total activities of the cellulases, peaked on day 5 [1, 7, 24]. These cellulases were applied to the enzymatic hydrolysis of SECS and the results are presented in Table 1. When using the same dosage, the cellulase from the parental strain Rut-C30 was found to have the serious cellobiose accumulation which negatively affected the enzymatic hydrolysis yield (red-colored in Table 1). Nevertheless, the enhanced production of β-glucosidases was proven capable of overcoming cellobiose accumulation and improving the enzymatic hydrolysis yield. From A8 to C4, the three cellulases reduced cellobiose concentrations to the neglectable level, lower than 1 g/L, and increased enzymatic hydrolysis yields to the desirable level, higher than 80%.

**Table 1:**
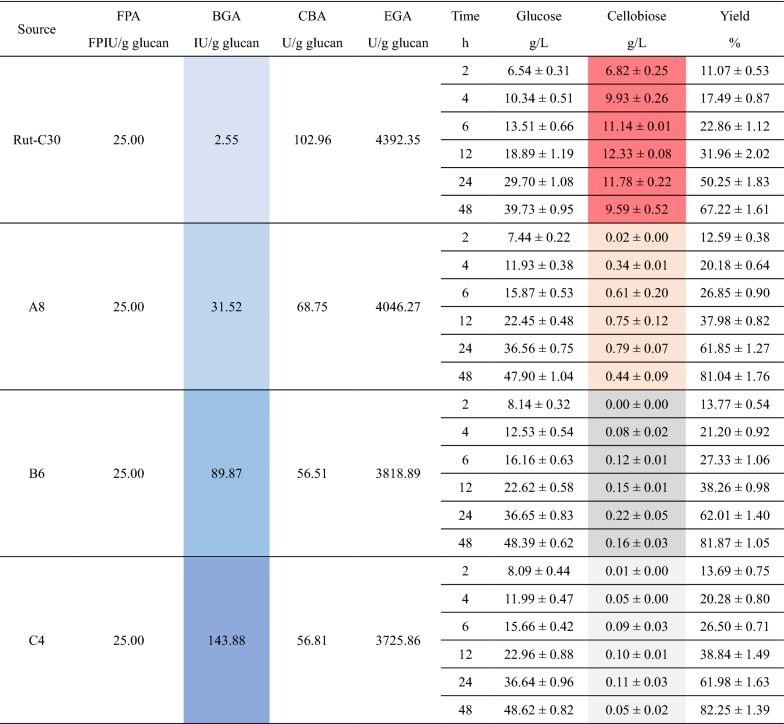
Results of the enzymatic hydrolysis by the cellulases from A8, B6, C4 and Rut-C30

The substrate used here was steam-exploded corn stover (SECS). All the cellulases were harvested after 5 days fermentation and they were used in the form of crude enzymes in the fermentation broths

The data about the cellulase activities were averages and the data about the enzymatic hydrolysis were presented in the form of average ± standard deviation

As BGA increased further, however, enzymatic hydrolysis yield no longer increased. This suggests that the BGA of A8 was high enough alleviate cellobiose accumulation and the BGAs beyond that were useless, unable to improve enzymatic hydrolysis yield any more. Hence, the wasted energy for overproduction of β-glucosidases was used for the enhanced production of CBH II because CBH components are the most important in the enzymatic hydrolysis of lignocelluloses [13, 18, 34] but *T. reesei* lacks of sufficient CBH II for efficient exo–exo synergism [6, 7].

In our previous studies [4, 7], FPA was enhanced as CBA was raised using the *T. reesei* strong promoter *Pcbh1*. This work adopted the same strategy to improve the CBH II production and BG production at the same for the simultaneous enhancement of the beta–exo synergism and exo–exo synergism in *Trichoderma reesei* cellulase.

### Simultaneous enhanced expression of *cbh2* gene and different *bgl1* genes

**Fig. 4:**
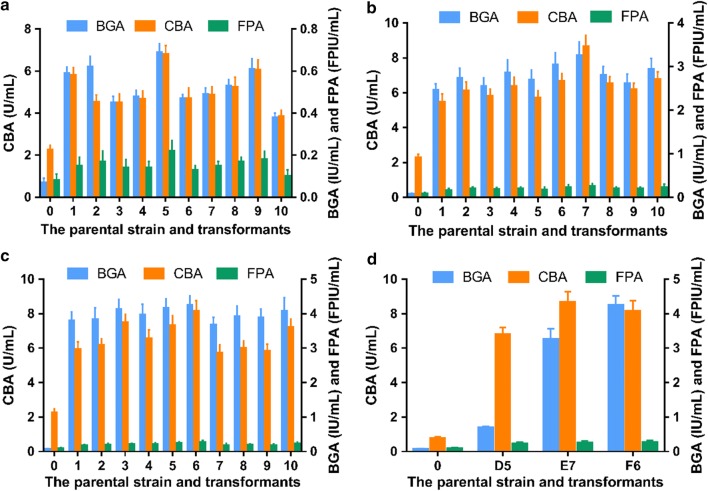
**a** BGAs, CBAs and FPAs of the 10 fastest-growing *T. reesei* transformants (1 to 10) harboring the strong expression cassettes of *T. reesei bgl1* gene and *cbh2* gene and the parental strain Rut-C30 (0); **b** BGAs, CBAs and FPAs of the 10 fastest-growing *T. reesei* transformants (1 to 10) harboring the strong expression cassettes of *A. aculeatus bgl1* gene and *T. reesei cbh2* gene and the parental strain Rut-C30 (0); **c** BGAs, CBAs and FPAs of the 10 fastest-growing *T. reesei* transformants (1 to 10) harboring the strong expression cassettes of *A. niger bgl1* gene and *T. reesei cbh2* gene and the parental strain Rut-C30 (0); **d** Comparison of the top *T. reesei* transformants D5, E7 and F6 from the transformants screening presented in **a**–**c**, using the parental strain Rut-C30 as the control (0)

The recombinant vector pCAMBIA1300-Ps-Trbgl1-T-Ps-Trcbh2-T (Fig. 1d) carrying the strong cassettes of *T. reesei bgl1* gene and *cbh 2* gene was transformed to *T. reesei*, producing 230 transformants. pCAMBIA1300 is the backbone, Ps the strong promoter of the *T. reesei cbh1* gene and its signal peptide sequence, Trbgl1 the *bgl1* gene of *T. reesei*, Trcbh2 the *cbh2* gene of *T. reesei*, and T the terminator of the *T. reesei cbh1* gene. After the two steps of transformant screening, 10 fastest-growing transformants were selected to compare their BGAs, CBAs and FPAs which are presented in Fig. 4a. The number 0 is *T. reesei* Rut-C30, and the numbers 1 to 10 are the *T. reesei* transformants. The ten *T. reesei* transformants D1–D10, cultured on the PDA agar plates without hygromycin B and were passed on from generation to generation for 10 times, were verified by genome PCR for the stable existence of the strong cassettes of *T. reesei bgl1* gene *and cbh 2* gene in their chromosomal DNA (data not shown).

It was found in Fig. 4a that the FPAs, CBAs and the BGAs of the transformants were substantially increased at the same time. Among the 10 fastest-growing transformants, the 5th transformant (named as D5) had the highest FPA, CBA and BGA which were 0.22 ± 0.05 FPIU/mL, 6.79 ± 0.42 U/mL and 0.69 ± 0.04 IU/mL after 48 h fermentation. They were 2.75, 3.00 and 9.86 times higher than the counterparts of Rut-C30, which were 0.08 ± 0.03 FPIU/mL, 2.26 ± 0.21 U/mL and 0.07 ± 0.02 IU/mL after 48 h fermentation.

The recombinant vector pCAMBIA1300-Ps-Aabgl1-T-Ps-Trcbh2-T (Fig. 1e) carrying the strong cassettes of *A. aculeatus bgl1* gene and *T. reesei cbh2* gene was transformed to *T. reesei*, producing 212 transformants. pCAMBIA1300 is the backbone, Ps the strong promoter of the *T. reesei cbh1* gene and its signal peptide sequence, Aabgl1 the *bgl1* gene of *A. aculeatus*, Trcbh2 the *cbh2* gene of *T. reesei*, and T the terminator of the *T. reesei cbh1* gene. After the two steps of transformant screening, 10 fastest-growing transformants were selected to compare their BGAs, CBAs and FPAs, as shown in Fig. 4b. The number 0 is *T. reesei* Rut-C30, and the numbers 1 to 10 are the *T. reesei* transformants. The ten *T. reesei* transformants E1–E10, cultured on the PDA agar plates without hygromycin B and were passed on from generation to generation for 10 times, were verified by genome PCR for the stable existence of the strong cassettes of *A. aculeatus bgl1* gene and *T. reesei cbh2* gene in their chromosomal DNA (data not shown).

It was found in Fig. 4b that the FPAs, CBAs and FPAs of the transformants were obviously improved. Among the 10 fastest-growing transformants, the 7th transformant (named as E7) had the highest FPA, CBA and BGA which were 0.25 ± 0.06 FPIU/mL, 8.65 ± 0.64 U/mL and 3.25 ± 0.31 IU/mL after 48 h fermentation. They were 3.13, 3.83 and 46.43 times higher than those of Rut-C30, which were 0.08 ± 0.03 FPIU/mL, 2.26 ± 0.21 U/mL and 0.07 ± 0.02 IU/mL after 48 h fermentation.

The recombinant vector pCAMBIA1300-Ps-Anbgl1-T-Ps-Trcbh2-T (Fig. 1f) carrying the strong cassettes of *A. niger bgl1* gene and *T. reesei cbh2* gene was transformed to *T. reesei*, producing 205 transformants. pCAMBIA1300 is the backbone, Ps the strong promoter of the *T. reesei cbh1* gene and its signal peptide sequence, Anbgl1 the *bgl1* gene of *A. niger*, Trcbh2 the *cbh2* gene of *T. reesei*, and T the terminator of the *T. reesei cbh1* gene. After the two steps of transformant screening, 10 fastest-growing transformants were selected to compare their BGAs, CBAs and FPAs, as shown in Fig. 4c. The number 0 is *T. reesei* Rut-C30, and the numbers 1 to 10 are the *T. reesei* transformants. The ten *T. reesei* transformants F1–F10, cultured on the PDA agar plates without hygromycin B and were passed on from generation to generation for 10 times, were verified by genome PCR for the stable existence of the strong cassettes of *A. niger bgl1* gene and *T. reesei cbh2* gene in their chromosomal DNA (data not shown).

It was found in Fig. 4c that the FPAs, CBAs and FPAs of the transformants were obviously improved. Among the 10 fastest-growing transformants, the 6th transformant (named as F6) produced the highest FPA, CBA and BGA which were 0.27 ± 0.05 FPIU/mL, 8.16 ± 0.60 U/mL and 4.24 ± 0.28 IU/mL after 48 h fermentation. They were 3.38-, 3.61- and 60.57-fold higher than those of Rut-C30, which were 0.08 ± 0.03 FPIU/mL, 2.26 ± 0.21 U/mL and 0.07 ± 0.02 IU/mL after 48 h fermentation.

Then the BGAs, BGAs and FPAs of the *T. reesei* transformants (D5, E7, F6) with CBH and BG being simultaneously enhanced were compared, as shown in Fig. 4d. It was found that the transformant F6 had the highest BGA. Next in line was E7 and D5. Compared to Rut-C30 (numbered as 0 in Fig. 4d), all the transformants’ BGAs were greatly improved. The BGAs may be dependent on the sources of *bgl1* genes because their enzymatic properties are different. Meanwhile, the CBAs were raised because the CBH II production was also enhanced by using the strong promoter *Pcbh1* of *T. reesei* [6, 7, 24]. Different from the results above where only the BGAs were greatly improved but the FPAs were slightly increased (Fig. 2d), here the FPAs were also greatly elevated mainly owing to the enhancement of the CBAs [7, 24]. After the CBA and BGA being enhanced simultaneously, the performance of the cellulase in enzymatic hydrolysis should be bettered in theory.

### Cellulase production with enhanced BGAs and CBAs and the application in enzymatic hydrolysis

**Fig. 5:**
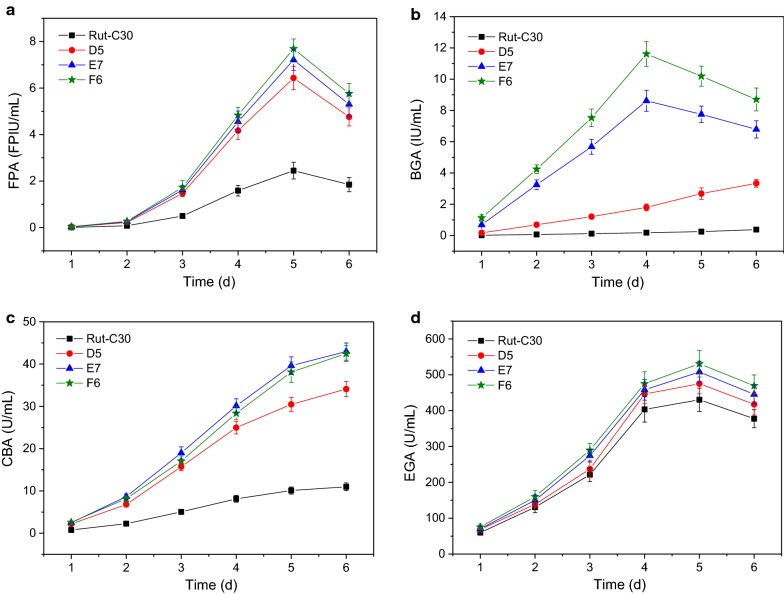
FPAs (**a**), BGAs (**b**), CBAs (**c**) and EGAs (**d**) in the fermentation broths obtained as a function of time from the *T. reesei* transformants D5, E7, F6, and parental strain Rut-C30 conducted in the 50 mL fermentation medium in 250 mL Erlenmeyer flasks incubated in incubatory shakers (170 rpm) at 30 °C on the first day and 28 °C since then

The time courses of the cellulase production by the *T. reesei* transformants D5, E7 and F6, as well as the control Rut-C30 are shown in Fig. 5. Unlike the observations in Fig. 3, the FPAs of the *T. reesei* transformants were significantly increased because of the enhancement of CBAs. The FPAs of D5, E7 and F6 had no big difference (Fig. 5a). This is probably because those transformants used the same strong expression cassette of *T. reesei cbh2* gene and nearly the same energy proportion of the strong promoter *Pcbh1* was used in the expression of the *cbh2* gene (here the three genes *cbh1*, *cbh2* and *bgl1* were sharing the strong promoter *Pcbh1*).

Figure 5b shows that the transformant F6 had the highest BGA, the second highest E7, the third D5. All the transformants’ BGAs were obviously increased and the differences in the BGAs were caused by the specific enzymatic activities of the different BGL I, which was discussed above. In addition, the BGAs of the transformants E7 and F6 peaked after 4 days fermentation while those of D5 and Rut-C30 ascended continuously. These observations are the same as those in Fig. 3b.

Opposite to the results of Fig. 3c, the CBAs here were significantly enhanced (Fig. 5c) because the strong promoter *Pcbh1* was used to strengthen the CBH II production which in line with the previous work [4, 7]. All of the CBAs demonstrated continuous increases during the fermentation processes because the strong promoter *Pcbh1* to manage the production of CBH I and CBH II at the same time continuously [7]. The transformant E7’s CBAs and F6’s CBAs were nearly the same and both of them were higher than D5’s CBAs, especially during the late fermentation period. The reason for this is unclear.

No large differences in EGAs were observed, as shown in Fig. 5d. The tiny increases in EGAs may be because that the improved CBAs did not cause negative effects on EGAs because they do not share same transcriptional factors, therefore no transcription titration effect happened, and/or that the increments of CBAs in cellulase disturbed the EGA determination, making it to some extent raised [7].

The cellulases produced by D5, E7, F6 and Rut-C30 were harvested after 5 days fermentation and applied to the enzymatic hydrolysis of SECS. The results are presented in Table 2. Compared to the cellulase from Rut-C30, the cellulases from D5, E7 and F6 exhibited much more excellent performances in the enzymatic hydrolysis. It is noteworthy, however, that the performance of the cellulase from D5 was to some extent inferior to those of the cellulases from E7 and F6. This must be because the proportions of BGA in those cellulases were not high enough for thoroughly eliminating cellobiose accumulation. The cellobiose concentrations manifested the explanation. The result here indicates that the enhanced co-expression of *cbh2* and *bgl1* both from *T. reesei* was not a good strategy for production of more robust cellulase. The key of this problem should be that the specific enzymatic activity of the *T. reesei* BGL I is too low, which had been proven by comparing the results of the related literatures [20–22].

**Table 2:**
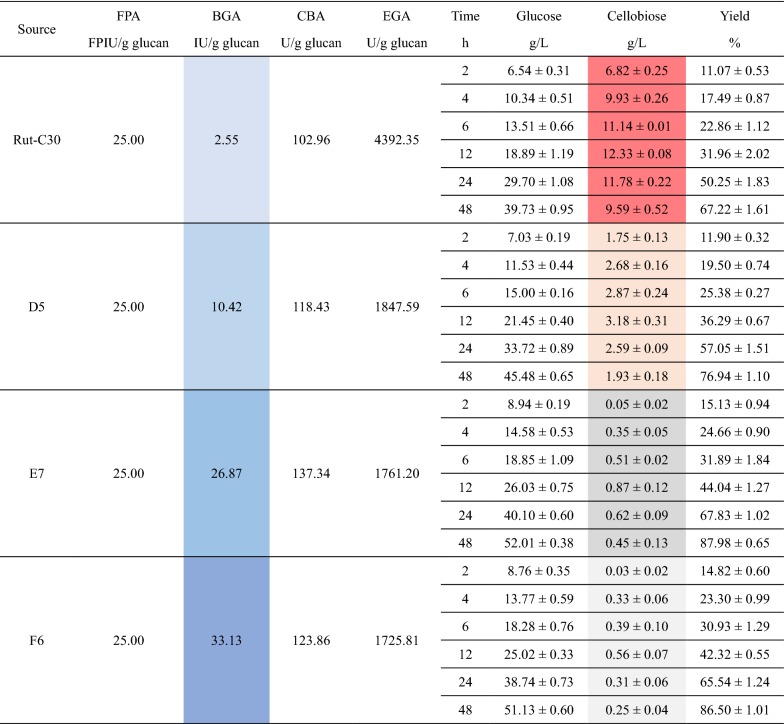
Results of the enzymatic hydrolysis by the cellulases from D5, E7, F6 and Rut-C30

The substrate used here was steam-exploded corn stover (SECS). All the cellulases were harvested after 5 days fermentation and they were used in the form of crude enzymes in the fermentation broths

The data about the cellulase activities were averages and the data about the enzymatic hydrolysis were presented in the form of average ± standard deviation

**Fig. 6:**
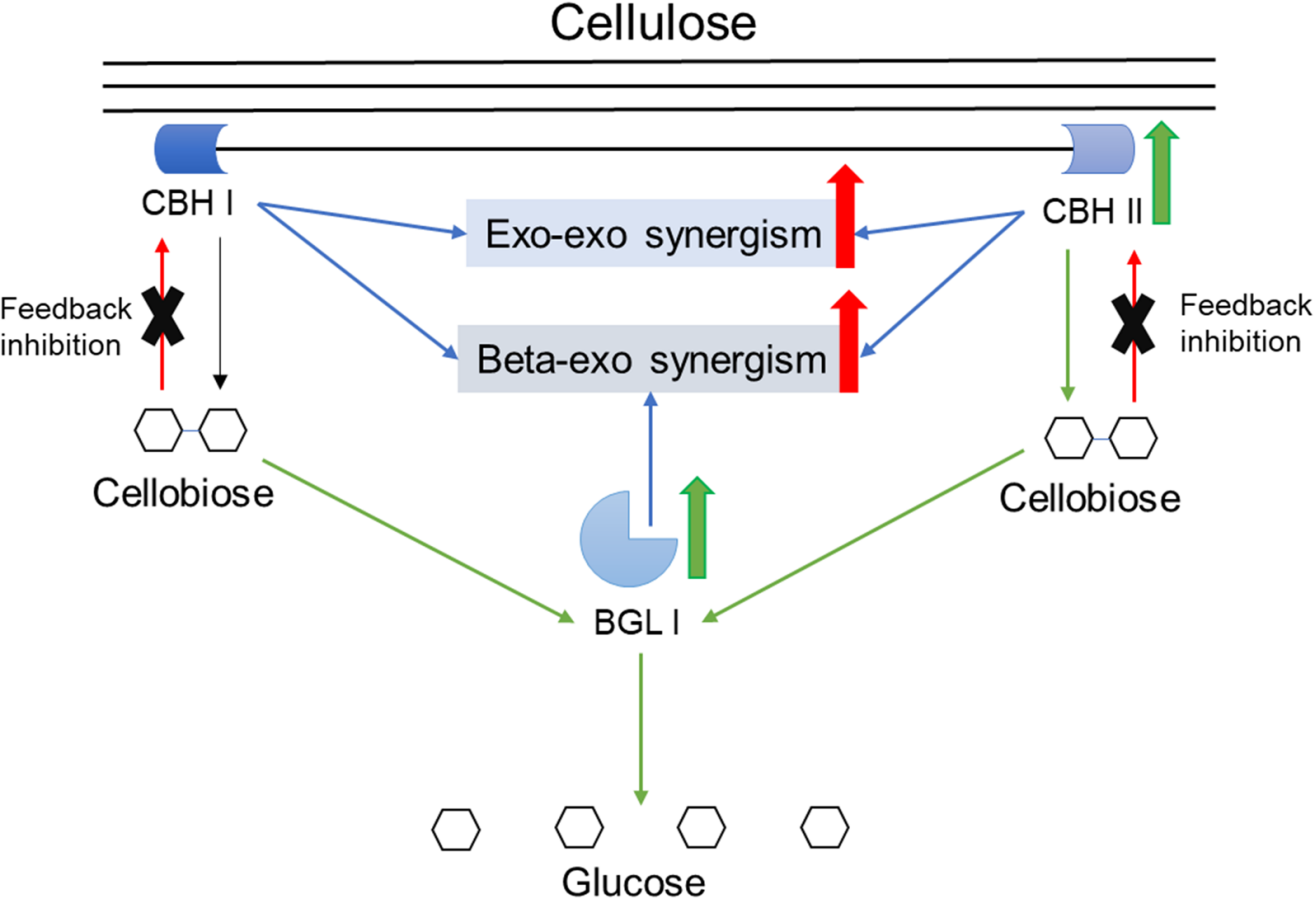
Simultaneous enhancement of the beta–exo synergism and exo–exo synergism of *T. reesei* cellulase

The cellulases from the transformants E7 and F6 outperformed because their CBAs and BGAs were enhanced at the same time, i.e. the two weaknesses of *T. reesei* cellulase were overcome meanwhile [4]. Not only the composition of *T. reesei* cellulase was improved, but also the high yields were achieved by the cellulases from E7 and F6, which were 87.98 ± 0.65% and 86.50 ± 1.01% respectively. The results prove that the beta–exo synergism and exo–exo synergism of *T. reesei* cellulase were enhanced simultaneously for more robust cellulase, as illustrated in Fig. 6, and thus the cellulose degrading capability was increased.

### Overall comparison of the different cellulases

The detailed information about the whole process from SECS to glucose in the context of on-site cellulase production [2, 37] is listed in Table 3. It was found that in general the cellulases from the transformants D5, E7 and F6 where the productions of CBH and BG were enhanced simultaneously were more efficient than the cellulases from the transformants A8, B6 and C4 where only the production of BG was enhanced. This makes sense because the formers had higher cellulase productivities and better synergisms that render cellulase more robust.

**Table 3:** Detailed information about the different cellulases

Source^a^	Rut-C30	A8	B6	C4	D5	E7	F6
FPA (FPIU/mL)	2.45	2.76	3.11	2.90	6.43	7.21	7.69
BGA (IU/mL)	0.25	3.48	11.18	16.69	2.68	7.75	10.19
CBA (U/mL)	10.09	7.59	7.03	6.59	30.46	39.61	38.10
EGA (U/mL)	430.45	446.71	475.07	432.20	475.20	507.93	530.86
g SECS for 1 FPIU cellulase	0.0122	0.0109	0.0096	0.0103	0.0047	0.0042	0.0039
Yield (%)	67.22	81.04	81.87	82.25	76.94	87.98	86.50
g/L glucose	39.73	47.90	48.39	48.62	45.48	52.01	51.13
FPIU cellulase for 1 g glucose	33.48	27.77	27.49	27.35	29.24	25.57	26.01
g SECS (total) for 1 g glucose	2.9537	2.3887	2.3317	2.3397	2.3352	2.0291	2.0573

^a^The cellulases produced by the parental strain Rut-C30, the transformants A8, B6, C4, D5, E7 and F6 respectively. All the cellulases were harvested after 5 d fermentation

Though the *T. reesei* transformant D5 had much higher FPA than the transformants A8, B6 and C4, the enzymatic hydrolysis yield by the cellulase from D5 was lower. Overall, however, the efficiency of the cellulase from D5 was similar to those of the cellulases from A8, B6 and C4. Totally 2.3352 g SECS was needed to produce 1 g glucose when using the cellulase from D5, which was more than those when using then cellulases from E7 and F6. The main problem was that the BG component was no longer enough when the strong promoter *Pcbh1* was co-expressing *T. reesei cbh2* and *bgl1* at the same time. The BGL I from *T. reesei* was not sufficiently strong in specific enzymatic activity is the crux of the matter because the transformants E7 and F6 in which the *bgl1* genes from *Aspergillus* species were expressed did not have the same problem.

The cellulases from the transformants E7 and F6 had the highest efficiencies because both of them had the highest FPAs and enzymatic hydrolysis yields. For these two transformants, only 2.0291 and 2.0573 g SECS were demanded to produce 1 g glucose, respectively. The former’s efficiency was a little higher than the latter’s, but not that different. This suggests that the transformants E7 and F6 were the best among all the *T. reesei* strains used in this work. Moreover, the lesson from the transformants D5 and A8 enlightens us that the transcriptional titration effect should be considered when using *Pcbh1* to express two or more genes at the same time and only using more robust cellulase component could save more energy for other cellulase component.

The results of the transformants E7 and F6 are better than those of the recombinant *T. reesei* in which only the production of CBH II was enhanced by using the strong promoter *Pcbh1* in the previous work [4]. This is because only one weakness of *T. reesei* cellulase was overcome and that recombinant *T. reesei* did still lack of β-glucosidase [24].

The strong promoter *Pcbh1* of *T. reesei* is not a master key capable of solving all the problems about the cellulase. More holistic angle of view should be introduced into the optimization of cellulase composition at the transcriptional level such as the transcriptional control engineering of the cellulase components as a whole by rearranging the promoters of different cellulase genes and quantitatively regulating their expression levels to push cellulase towards perfection in the context of real-world applications. Therefore, further work should be done to extend more and dig deeper in this research field.

## Conclusions

Enhanced co-expression of the β-glucosidase gene from *A. aculeatus* or *A. niger* and the cellobiohydrolase II gene of *T. reesei* using the strong promoter *Pcbh1* was found to be the best strategy in overcoming the two weaknesses of *T. reesei* cellulase, deficiencies in β-glucosidase and CBH II. This strategy improved cellulase production by *T. reesei* and optimized the cellulase composition to better the synergisms and increase cellulose degrading capability. The enzymatic hydrolysis yield and the efficiency of the process from SECS to glucose in the context of on-site cellulase production were greatly improved here by simultaneously strengthening the beta–exo synergism and exo–exo synergism in *T. reesei* cellulase. This work serves as a modest spur to optimize the *T. reesei* cellulase at the transcriptional level by transcriptional control engineering.

## Abbreviations

AMT: *Agrobacterium tumefaciens*-mediated transformation
BG: β-glucosidase
BGA: β-glucosidase activity
BGL I: β-glucosidase I
CBA: cellobiohydrolase activity
CBH: cellobiohydrolase
CBH II: cellobiohydrolase II
EG: endoglucanase
EGA: endoglucanase activity
FPA: filter paper activity
FPIU: international unit of filter paper activity
HPLC: high performance liquid chromatography
IM: induction medium
IU: international unit
IUPAC: International Union of Pure and Applied Chemistry
LB: Luria–Bertani
MCC: microcrystalline cellulose
PDA: potato dextrose agar
SECS: steam exploded corn stover

## References

[1] Fang H, Zhao C, Song XY (2010). Optimization of enzymatic hydrolysis of steam-exploded corn stover by two approaches: response surface methodology or using cellulase from mixed cultures of *Trichoderma reesei* RUT-C30 and *Aspergillus niger* NL02. Bioresour Technol.

[2] Zhao C, Zou Z, Li J, Jia H, Liesche J, Fang H, Chen S (2017). A novel and efficient bioprocess from steam exploded corn stover to ethanol in the context of on-site cellulase production. Energy.

[3] Garvey M, Klose H, Fischer R, Lambertz C, Commandeur U (2013). Cellulases for biomass degradation: comparing recombinant cellulase expression platforms. Trends Biotechnol.

[4] Zhao C, Deng L, Fang H (2018). Mixed culture of recombinant *Trichoderma reesei* and *Aspergillus niger* for cellulase production to increase the cellulose degrading capability. Biomass Bioenergy.

[5] Bischof RH, Ramoni J, Seiboth B (2016). Cellulases and beyond: the first 70 years of the enzyme producer *Trichoderma reesei*. Microb Cell Fact.

[6] Fang H, Xia L (2015). Heterologous expression and production of *Trichoderma reesei* cellobiohydrolase II in *Pichia pastoris* and the application in the enzymatic hydrolysis of corn stover and rice straw. Biomass Bioenergy.

[7] Fang H, Xia L (2013). High activity cellulase production by recombinant *Trichoderma reesei* ZU-02 with the enhanced cellobiohydrolase production. Bioresour Technol.

[8] Kim IJ, Jung JY, Lee HJ, Park HS, Jung YH, Park K, Kim KH (2015). Customized optimization of cellulase mixtures for differently pretreated rice straw. Bioprocess Biosyst Eng.

[9] Qian Y, Zhong L, Gao J, Sun N, Wang Y, Sun G, Qu Y, Zhong Y (2017). Production of highly efficient cellulase mixtures by genetically exploiting the potentials of *Trichoderma reesei* endogenous cellulases for hydrolysis of corncob residues. Microb Cell Fact.

[10] Khare SK, Pandey A, Larroche C (2015). Current perspectives in enzymatic saccharification of lignocellulosic biomass. Biochem Eng J.

[11] Sanchez C (2009). Lignocellulosic residues: biodegradation and bioconversion by fungi. Biotechnol Adv.

[12] Goacher RE, Selig MJ, Master ER (2014). Advancing lignocellulose bioconversion through direct assessment of enzyme action on insoluble substrates. Curr Opin Biotechnol.

[13] Zhou J, Wang YH, Chu J, Luo LZ, Zhuang YP, Zhang SL (2009). Optimization of cellulase mixture for efficient hydrolysis of steam-exploded corn stover by statistically designed experiments. Bioresour Technol.

[14] Saloheimo M, Pakula TM (2012). The cargo and the transport system: secreted proteins and protein secretion in *Trichoderma reesei* (*Hypocrea jecorina*). Microbiology.

[15] Singhania RR, Sukumaran RK, Patel AK, Larroche C, Pandey A (2010). Advancement and comparative profiles in the production technologies using solid-state and submerged fermentation for microbial cellulases. Enzyme Microb Technol.

[16] Gupta VK, Steindorff AS, de Paula RG, Silva-Rocha R, Mach-Aigner AR, Mach RL, Silva RN (2016). The post-genomic era of *Trichoderma reesei*: what’s next?. Trends Biotechnol.

[17] Fang H, Zhao C, Song X-Y, Chen M, Chang Z, Chu J (2013). Enhanced cellulolytic enzyme production by the synergism between *Trichoderma reesei* RUT-C30 and *Aspergillus niger* NL02 and by the addition of surfactants. Biotechnol Bioprocess Eng.

[18] Billard H, Faraj A, Lopes Ferreira N, Menir S, Heiss-Blanquet S (2012). Optimization of a synthetic mixture composed of major *Trichoderma reesei* enzymes for the hydrolysis of steam-exploded wheat straw. Biotechnol Biofuels.

[19] Culbertson A, Jin M, da Costa Sousa L, Dale BE, Balan V (2013). In-house cellulase production from AFEX™ pretreated corn stover using *Trichoderma reesei* RUT C-30. RSC Adv.

[20] Nakazawa H, Kawai T, Ida N, Shida Y, Kobayashi Y, Okada H, Tani S, Sumitani J, Kawaguchi T, Morikawa Y, Ogasawara W (2012). Construction of a recombinant *Trichoderma reesei* strain expressing *Aspergillus aculeatus* beta-glucosidase 1 for efficient biomass conversion. Biotechnol Bioeng.

[21] Zhang J, Zhong Y, Zhao X, Wang T (2010). Development of the cellulolytic fungus *Trichoderma reesei* strain with enhanced beta-glucosidase and filter paper activity using strong artificial cellobiohydrolase 1 promoter. Bioresour Technol.

[22] Wang B, Xia L (2011). High efficient expression of cellobiase gene from *Aspergillus niger* in the cells of *Trichoderma reesei*. Bioresour Technol.

[23] Chen M, Zhao J, Xia L (2008). Enzymatic hydrolysis of maize straw polysaccharides for the production of reducing sugars. Carbohydr Polym.

[24] Fang H, Xia L (2015). Cellulase production by recombinant *Trichoderma reesei* and its application in enzymatic hydrolysis of agricultural residues. Fuel.

[25] Yang J, Zhang X, Yong Q, Yu S (2010). Three-stage hydrolysis to enhance enzymatic saccharification of steam-exploded corn stover. Bioresour Technol.

[26] Kolasa M, Ahring BK, Lubeck PS, Lubeck M (2014). Co-cultivation of *Trichoderma reesei* RutC30 with three black *Aspergillus* strains facilitates efficient hydrolysis of pretreated wheat straw and shows promises for on-site enzyme production. Bioresour Technol.

[27] Kubicek-Pranz EM, Gruber F, Kubicek CP (1991). Transformation of *Trichoderma reesei* with the cellobiohydrolase II gene as a means for obtaining strains with increased cellulase production and specific activity. J Biotechnol.

[28] Te’o VSJ, Cziferszky AE, Bergquist PL, Nevalainen KMH (2000). Codon optimization of xylanase gene xynB from the thermophilic bacterium *Dictyoglomus thermophilum* for expression in the filamentous fungus *Trichoderma reesei*. FEMS Microbiol Lett.

[29] Zhao C, Fang H, Chen S (2017). Single cell oil production by *Trichosporon cutaneum* from steam-exploded corn stover and its upgradation for production of long-chain α, ω-dicarboxylic acids. Biotechnol Biofuels.

[30] Michielse CB, Hooykaas PJ, van den Hondel CA, Ram AF (2008). Agrobacterium-mediated transformation of the filamentous fungus *Aspergillus awamori*. Nat Protoc.

[31] Mandels M, Medeiros JE, Andreotti RE, Bissett FH (1981). Enzymatic hydrolysis of cellulose: evaluation of cellulase culture filtrates under use conditions. Biotechnol Bioeng.

[32] Ghose TK (1987). Measurement of cellulase activities. Pure Appl Chem.

[33] Miller GL (1959). Use of dinitrosalicylic acid reagent for determination of reducing sugar. Anal Chem.

[34] Gao D, Chundawat SPS, Krishnan C, Balan V, Dale BE (2010). Mixture optimization of six core glycosyl hydrolases for maximizing saccharification of ammonia fiber expansion (AFEX) pretreated corn stover. Bioresour Technol.

[35] Nevalainen H, Peterson R (2014). Making recombinant proteins in filamentous fungi- are we expecting too much?. Front Microbiol.

[36] Landowski CP, Huuskonen A, Wahl R, Westerholm-Parvinen A, Kanerva A, Hanninen AL, Salovuori N, Penttila M, Natunen J, Ostermeier C (2015). Enabling low cost biopharmaceuticals: a systematic approach to delete proteases from a well-known protein production host *Trichoderma reesei*. PLoS ONE.

[37] Zhao C, Zou Z, Li J, Jia H, Liesche J, Chen S, Fang H (2018). Efficient bioethanol production from sodium hydroxide pretreated corn stover and rice straw in the context of on-site cellulase production. Renew Energy.

